# Isolation of a New *Chlamydia* species from the Feral Sacred Ibis (*Threskiornis aethiopicus*): *Chlamydia* *ibidis*

**DOI:** 10.1371/journal.pone.0074823

**Published:** 2013-09-20

**Authors:** Fabien Vorimore, Ru-ching Hsia, Heather Huot-Creasy, Suzanne Bastian, Lucie Deruyter, Anne Passet, Konrad Sachse, Patrik Bavoil, Garry Myers, Karine Laroucau

**Affiliations:** 1 University Paris-Est, Anses, Animal Health Laboratory, Bacterial Zoonoses Unit, Maisons-Alfort, France; 2 University of Maryland, Core Imaging Facility, Baltimore, Maryland, United States of America; 3 University of Maryland, Institute for Genome Sciences, Baltimore, Maryland, United States of America; 4 Université Nantes Angers, Le Mans, Nantes, France; 5 ONIRIS, UMR1300, Bio-agression, Epidémiologie et Analyse de Risques, Nantes, France; 6 INRA, UMR1300, Bio-agression, Epidémiologie et Analyse de Risques, Nantes, France; 7 ONIRIS, Center for Wildlife and Ecosystem Health, Nantes, France; 8 Institute of Molecular Pathogenesis, Friedrich-Loeffler-Institut, Jena, Germany; 9 University of Maryland, Department of Microbial Pathogenesis, Baltimore, Maryland, United States of America; University of Lausanne, Switzerland

## Abstract

Investigations conducted on feral African Sacred Ibises ( *Threskiornis* *aethiopicus* ) in western France led to the isolation of a strain with chlamydial genetic determinants. Ultrastructural analysis, comparative sequence analysis of the 16S rRNA gene, *omp*A, and of a concatenate of 31 highly conserved genes, as well as determination of the whole genome sequence confirmed the relatedness of the new isolate to members of the *Chlamydiaceae*, while, at the same time demonstrating a unique position outside the currently recognized species of this family. We propose to name this new chlamydial species *Chlamydia* *ibidis* *.*

## Introduction

As they are relatively easily kept in captivity, African Sacred Ibises have been introduced into many zoological parks around the world. In the 1990s, some of these birds escaped from a zoological park in Brittany (western France), where they had been allowed to fly unrestrained. Subsequently, a feral population established itself and disseminated along the wetlands of the French Atlantic coast. The feral population steadily increased to more than 5,000 birds in 2006 [1]. Major concerns on its possible impact on biodiversity arose from the birds’ predatory behaviour and ecological niche, as well as their potential role as a reservoir for the transmission of pathogens [2].

Avian chlamydiosis has been shown to occur in more than 465 avian species, including domestic, companion and wild birds [3]. *Chlamydia psittaci* is the most prominent chlamydial agent encountered in *Aves*. The family *Chlamydiaceae* currently comprises one genus *Chlamydia* and eight other species: *trachomatis*, *suis, muridarum*, *pneumoniae*, *abortus*, *caviae*, *felis*, and *pecorum* [4].

Laboratory diagnosis of chlamydial infections has undergone a remarkable methodological evolution in the past two decades [5]. Notably, multiple PCR-based detection methods that allow rapid detection of all species of the family *Chlamydiaceae* have been developed. Based on these methods, new and hitherto non-classified chlamydial agents have been recently described [6-12].

In accordance with international conventions, the French authorities have allowed culling operations since 2007 in order to reduce the population of this invasive bird. As ibises were repeatedly observed to feed on open-air duck breeding premises [13], where avian chlamydiosis is highly prevalent [14-16], investigations were therefore conducted on ibises shot during official culling operations.

Here, we report the results of a study conducted on ibises shot during the official culling operations carried out in western France in 2009 and 2010.

## Materials and Methods

### Samples

The Sacred Ibis is a feral species (origin: zoos) in Western and Southern France. It is considered in France and Europe of concern because of its invasive capacity (see www.europe-aliens.org). France has taken regulations for its destruction, by means of annual County Regulation Acts, that authorize destruction, with details on the protocol and laboratories authorized for analysis. The operations of destruction are ongoing since 2008. From March 2009 to July 2010, the 70 feral birds used in this study were shot by governmental environmental police agents (ONCFS), with precaution not to harm or scare other protected species, as their habitats are protected wetlands. These 70 birds were members of a group that was repeatedly observed to visit duck breeding premises, wetlands, landfills and ruminant manure. Animals that were not dead after being shot were put down by vertebral dislocation. Sample collection was done on dead animals only. For this study, the protocol of destruction was not submitted to an ethical committee, but the regulations were taken after discussion with local stakeholders and wildlife protection non-governmental organizations, in conformity with French, European and International laws and treaties. The transfer of duplicate cloacal swabs from these birds to the research laboratories where this study was performed was authorized by way of administrative decrees (Prefecture du Morbihan, "arrets" dated 23 February 2009 and 09 July 2010). The first swab was stored dry, to be subjected to DNA extraction, while the second swab was stored in SPG storage buffer [17] at 80°C, later to be used for inoculation of chicken eggs. Key data are summarized in Table **1**.

**Table 1:** Examination of samples from feral Sacred Ibis populations in 2009 and 2010.

**ID**	**Date of capture**	**23S-rtPCR *Chlamydiaceae***			***incA*-rtPCR *C. psittaci***	**Cell culture isolation**
		**No. of +ve samples**	**mean Ct**		**No. of +ve samples**	
09-0370	10/03/2009	0/21	-		-	-
09-0468	30/03/2009	1/15	30.78		0/1	0/1
10-1398	01/07/2010	7/34	34.93		1/7	3/7
Total		8/70			1/8	3/8

### Direct detection of *Chlamydiaceae* DNA from avian samples

The dry panel of cloacal swabs was subjected to DNA extraction using a QIAamp DNA Mini Kit, following the buccal swab protocol (Qiagen, Courtaboeuf, France). DNA was eluted with 150 µl of AE buffer and stored at -20°C before analysis.

A *Chlamydiaceae*-specific real-time PCR targeting the 23S rRNA gene was used in this study [18]. The protocol includes primers Ch23S-F (5'-CTGAAACCAGTAGCTTATAAGCGGT-3'), Ch23S-R (5'-ACCTCGCCGTTTAACTTAACTCC-3'), and probe Ch23S-p (FAM-5’-CTCATCATGCAAAAGGCACGCCG-3’-TAMRA). Each reaction mix contained 2 µl sample DNA template, 10 µl of Universal Master mix 2X (Applied Biosystems), 0.5 µl of each primer (25 µM) and 2 µl of the probe (1 µM), and 5 µl deionized water. The temperature-time profile was 95°C 10 min, 50 cycles of 95°C 15 s, 60°C 60 s. An internal, positive control was included in each sample to test for the presence of PCR inhibitors (Exogenous internal positive control, TaqMan, Life technologies).

### Inoculation of embryonated chicken eggs

For culture, suspensions of cloacal swabs stored in storage buffer at -80°C were thawed, transferred into sterile Eppendorf tubes and centrifuged at 10,000 rpm for 5 min. Supernatants were transferred into a new sterile tube. Antibiotic solution containing 1 mg/ml of vancomycin, 1 mg/ml of streptomycin, 1 mg/ml of kanamycin and 1000 units/ml of nystatin was added to the supernatant and to the resuspended pellet, which were then incubated at 37°C for 2 h before inoculation.

Yolk sacs of 7 day-old embryonated eggs were inoculated with 0.2 ml per egg, and 5 eggs per sample. For each set of inoculations, 3 eggs were inoculated with *C. psittaci* Loth as a positive control, and 3 other eggs were kept separately as non-infected controls. Eggs were incubated at 38°C and observed daily. Vitellus membranes were collected, then analysed using either a MIF test, i.e. a direct immunofluorescence assay ( *Chlamydia* direct IF, BioMérieux, Marcy l’Etoile, France) for samples collected in 2009, or *Chlamydiaceae*-specific real-time PCR for samples collected in 2010.

### DNA-based characterization

#### *C. psittaci*-specific real-time PCR

The *inc*A-based real-time PCR assay specific for *C. psittaci* was conducted as previously described [19].

#### Amplifications for DNA sequencing

Primers used for the partial amplification of *omp*A and 16S rRNA genes are listed in Table **S1**. PCR was performed in a total volume of 50 µl containing 4 µl DNA template, 5 µl 10X PCR reaction buffer, 2 U Hot start Taq DNA polymerase (Qiagen, Courtaboeuf, France), 400 µM of each deoxynucleotide triphosphate, and 0.5 µM of each flanking primer.

#### DNA sequencing

PCR-amplified segments of *omp*A and the 16S rRNA gene were purified using the QIAquick PCR Purification Kit (Qiagen, Courtaboeuf, France). DNA sequencing was performed at MWG Biotech France (Roissy, France).

Nucleotide sequences have been deposited under GenBank nos. HQ662953 (10-1398/28), HQ662954 (10-1398/11), HQ662955 (10-1398/6) for the 16S rRNA gene, and nos. HQ662949 (10-1398/28), HQ662950 (08-1274_Flock21), HQ662951 (10-1398/11), HQ662952 (10-1398/6) for *omp*A.

#### o*mp*A and 16S rRNA gene sequence analysis

o*mp*A and 16S rRNA gene sequences determined in this study were combined in an alignment with previously published sequences that were relevant for phylogenetic and epidemiological considerations. Sequence data were analyzed using the Bionumerics software package version 4.6 (Applied-Maths, Saint-Martens-Latem, Belgium) as a character dataset. Cluster analysis was conducted using the categorical parameter and the UPGMA coefficient.

#### Genome sequence analysis

Parallel pyrosequencing of the 10-1398/6 isolate was performed using a 454 Genome Sequencer FLX instrument with GS FLX Titanium series reagents. 449,422 sequence reads were obtained (mean length=342,9 bp; median length= 97,0 bp; total bases=154,112,763 bp). *De novo* assembly yielded a draft genome of 20 contigs. Four contigs mapped and were ordered to reference strain *C. psittaci* 6BC, in order to generate a pseudomolecule spanning 1,146,174 bp. The draft genome was annotated using the University of Maryland Institute for Genome Sciences annotation engine (http://ae.igs.umaryland.edu/cgi/index.cgi). Using this annotated draft sequence, the concatenated sequences of 31 conserved genes (*dna*G, *frr*, *inf*C, *nus*A, *pgk*, *pyr*G, *rpl*A, *rpl*B, *rpl*C, *rpl*D, *rpl*E, *rpl*F, *rpl*K, *rpl*L, *rpl*M, *rpl*N, *rpl*P, *rpl*S, *rpl*T, *rpm*A, *rpo*B, *rps*B, *rps*C, *rps*E, *rps*I, *rps*J, *rps*K, *rps*M, *rps*S, *smp*B, and *tsf*) [20] were assembled and compared with the corresponding gene sequences from all other members of the *Chlamydiaceae* family. Predicted genes were compared by BLAST against the complete set of genes from other chlamydial genomes using an E-value cutoff of 10^−5^. Synteny and BLAST Score Ratio (BSR) analyses were performed as previously described [21]. Whole nucleotide sequence of 10-1398/6 has been deposited under GenBank no APJW00000000 (Bioproject PRJNA188464).

### Electron microscopy

Vero cells were infected with a homogenate from vitellus membranes previously infected with isolate 10-1398/6. Infected cells were harvested at 48 hours post-infection. After washing, cells were fixed in 2% paraformaldehyde and 2.5% glutaraldehyde, scrapped off the culture plate and enrobed in agarose. Agarose containing infected cells was trimmed into ~1mm^3^ cubes, post-fixed with 1% osmium tetroxide, washed and *en bloc* stained with 1% uranyl acetate in water. Specimens were then washed and dehydrated using 30%, 50%, 70%, 90% and 100% ethanol in series, 10 minutes each. This was followed by two more 100% ethanol washes and infiltration with increasing concentrations of Spurr resin (Electron Microscopy Sciences, PA). After two exchanges of pure resin, specimens were embedded in Spurr resin and polymerized at 60°C overnight. Silver colored ultrathin (~70nm) sections were cut and collected using a Leica UC6 ultramicrotome (Leica Microsystems, Inc., Bannockburn, IL), counterstained with uranyl acetate and lead, and examined in a transmission electron microscope (Tecnai T12, FEI) operated at 80 kV. Digital images were acquired using an AMT bottom mount CCD camera and AMT600 software (Advanced Microscopy Techniques, MA).

## Results

### Detection and isolation of chlamydiae from the African Sacred Ibis, *Threskiornis* *aethiopicus*

In the present work, examination of cloacal swabs collected from 70 birds using *Chlamydiaceae*-specific real-time PCR demonstrated that 11% (8/70) of the apparently healthy birds had chlamydiae in their digestive tract (Table **1**). When re-tested with a *C. psittaci inc*A-specific real-time PCR, only one of the 8 positive Ibis samples remained positive: sample 10-1398/28. The Ct values indicated different levels of excretion among individual birds, three of which qualified as high excreters (Ct < 30), i.e. 09-468/3, 10-1398/6, 10-1398/28.

Duplicates of the *Chlamydiaceae* PCR-positive dry swabs, which had been stored in SPG medium, were inoculated into embryonated chicken eggs. Chlamydiae were successfully cultured from 3 samples: 10-1398/6, 10-1398/11 and 10-1398/28 (Table **1**). Isolation was unsuccessful from sample 09-468/3, although it had a high Ct value.

### Molecular characterization of the *T* *. aethiopicus* chlamydial isolates

**Figure 1:**
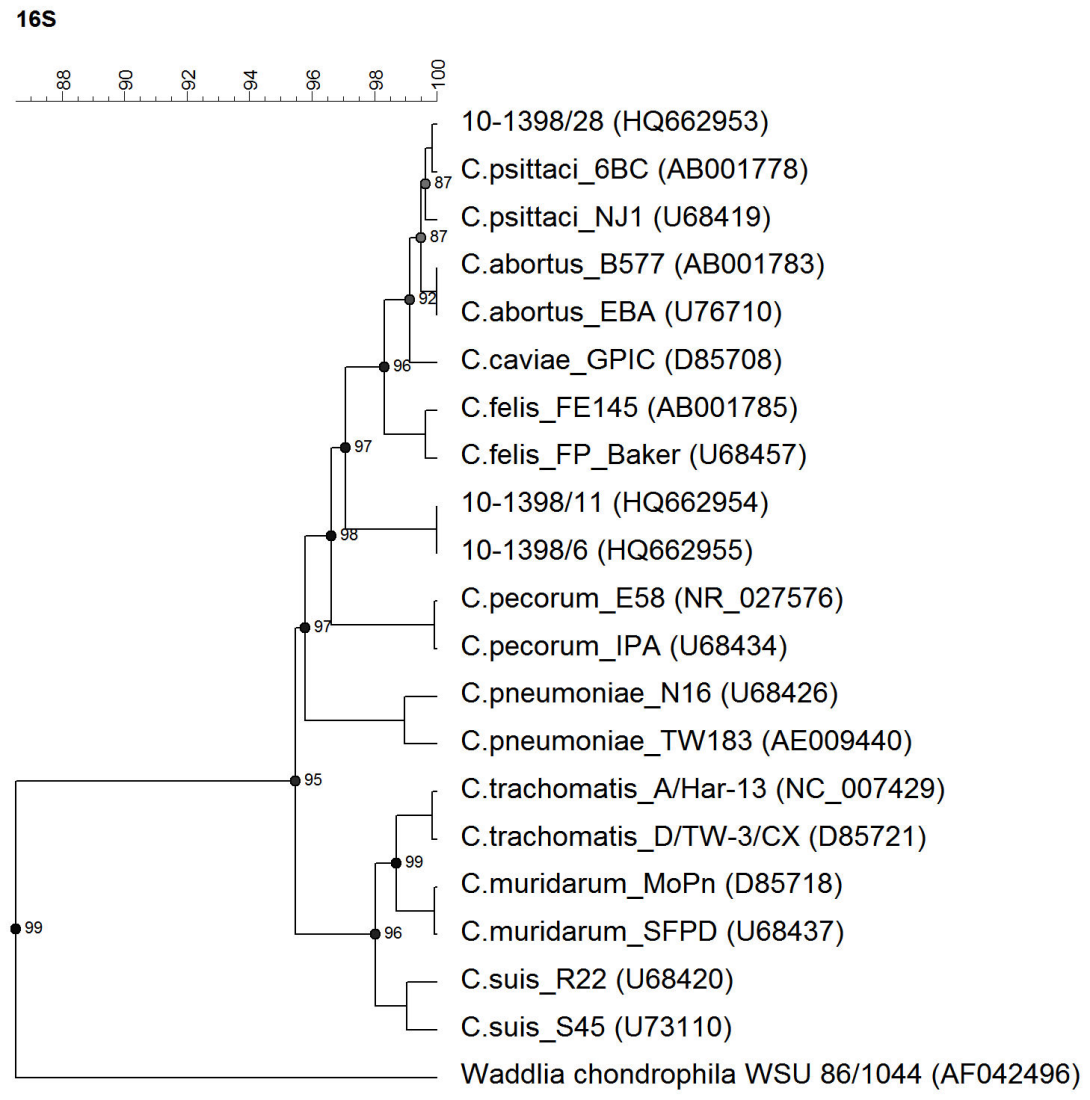
Dendrogram based on the analysis of the nearly complete 16S rRNA gene sequences (about 1350 nt) from the Ibis isolates (10-1398/6 and 10-1398/11) and from the type strains of nine *Chlamydiaceae*. The dendrogram was constructed by UPGMA method from a similarity matrix calculated by pairwise alignment. Branch quality was calculated by cophenetic correlation. Horizontal distances correspond to genetic distances expressed in percentage of sequence similarity.

The 16S rRNA and *omp*A gene sequences were obtained to establish the identity of the 3 chlamydial isolates and to indicate their taxonomic position. The 16S rRNA gene sequences of isolates 10-1398/6 and 10-1398/11 were identical. Alignment with representative sequences of all *Chlamydia* spp. established the position of these isolates within the family *Chlamydiaceae*, but outside the existing species (Figure **1**). Sequence similarity measurements listed in Table **S2** confirmed the distinct genetic position of these two isolates. However, the 16S rRNA gene sequence of isolate 10-1398/28 was highly homologous to that of the *C. psittaci* reference strain 6BC (99.85% similarity) (Table **S2**).

BLAST analysis of the *omp*A sequences confirmed that isolate 10-1398/28 is closely related to *C. psittaci* strain GD (genotype C) whereas the 10-1398/6 and 10-1398/11 isolates displayed lower similarity to *omp*A of other *Chlamydia* spp. (up to 76% versus *C. felis* and *C. psittaci*). This further suggested that these two isolates held a phylogenetic position outside the established chlamydial species. The *omp*A-based dendrogram in Figure **S1** confirms that these two isolates form a separate cluster.

### Ultrastructural analysis of inclusions generated upon infection with isolate 10-1398/6

**Figure 2:**
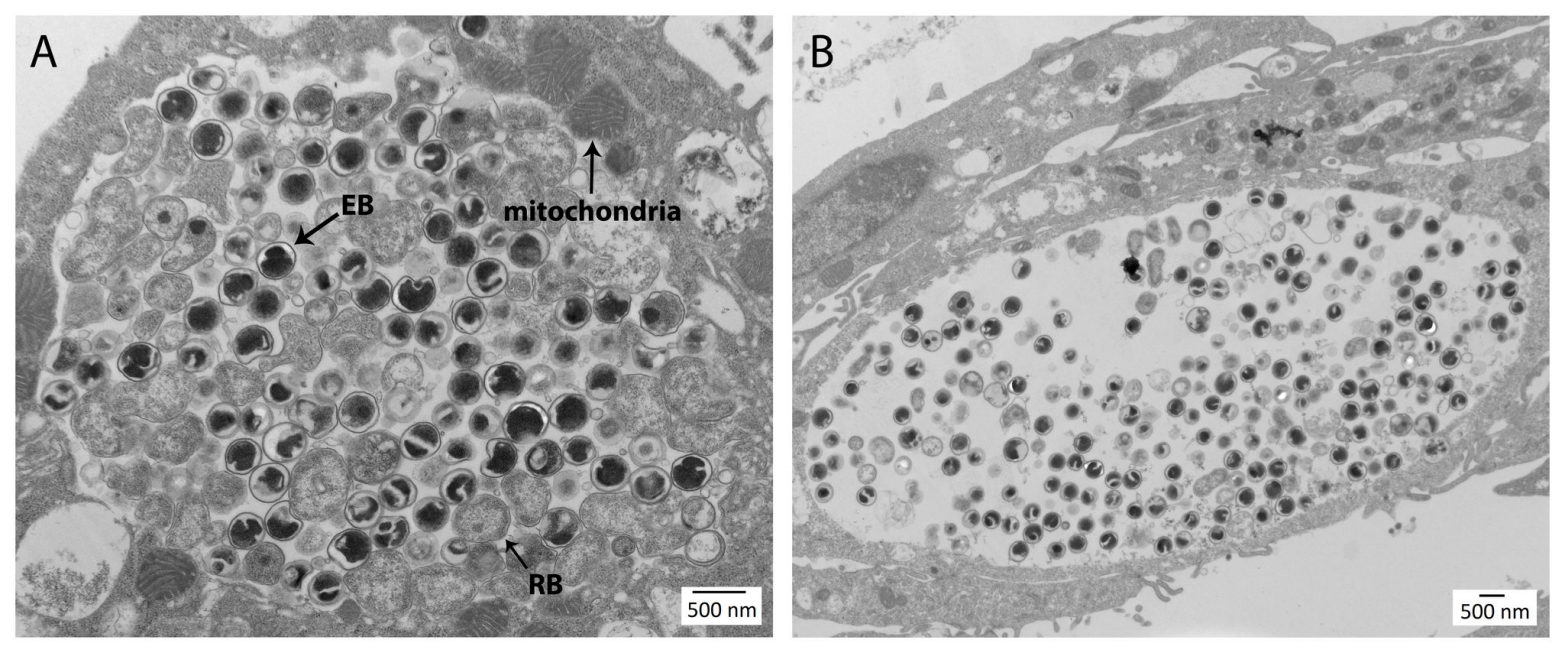
Ultrastructural analysis of inclusions generated upon infection of Vero cells with isolate 10-1398/6 at 48h post infection. Two typical inclusions were observed, less (A) or more (B) mature.

Vero cells infected with isolate 10-1398/6 displayed inclusions at different stages of development similar in ultrastructure to inclusions generated upon infection with *C. psittaci* [22]. Figure **2** displays two typical inclusions observed at 48 hours post-infection. In the less mature, densely packed inclusion shown in Figure **2A**, developmental forms corresponding to elementary bodies (EBs) and reticulate bodies (RBs) were observed in similar numbers. EBs were typically spherical with a dense nucleoid, clear periplasm and thickened cell wall as their *C. psittaci* counterparts [22]. However, some EBs of isolate 10-1398/6 appeared somewhat larger in diameter than *C. psittaci* EBs (350-400 nm vs. 300-350 nm [22]). Moreover, the condensed nucleoid appeared lobar or folded (as a “wallet”) in many EBs of isolate 10-1398/6, a distinguishing feature that was not observed in micrographs of *C. psittaci* EBs prepared under the same conditions (not shown). RBs of isolate 10-1398/6 were larger, typically pleiomorphic, with a cell wall that appeared thinner by TEM than that of EBs, and had a loosely distributed, somewhat granular cytoplasm lacking a condensed nucleoid. In the few inclusions that we observed at this developmental stage, most RBs were associated with or near the inclusion membrane, consistent with the proposed contact-dependent model of chlamydial development [23]. Intermediate bodies that display a smaller condensed nucleoid and a partially cleared cytoplasm were also apparent in small numbers. The more mature inclusion of isolate 10-1398/6 shown in Figure **2B** was significantly less packed and contained mostly EBs. Both inclusions also displayed evidence of fusion with cytolosic host vesicles and contained apparent RB ghosts and many smaller vesicles. Finally, the more actively replicating inclusion shown in Figure **2A** was selectively associated with mitochondria, particularly in areas of the inclusion membrane that are associated with RBs on the inclusion side. Although this is consistent with the reported preferred association of the *C. psittaci* inclusion with mitochondria [24], observation of a larger number of inclusions at this developmental stage would be required to establish statistical significance.

### Phylogenetic characterization of a new *Chlamydia* species from *T* *. aethiopicus* : *Chlamydia* *ibidis*

**Figure 3:**
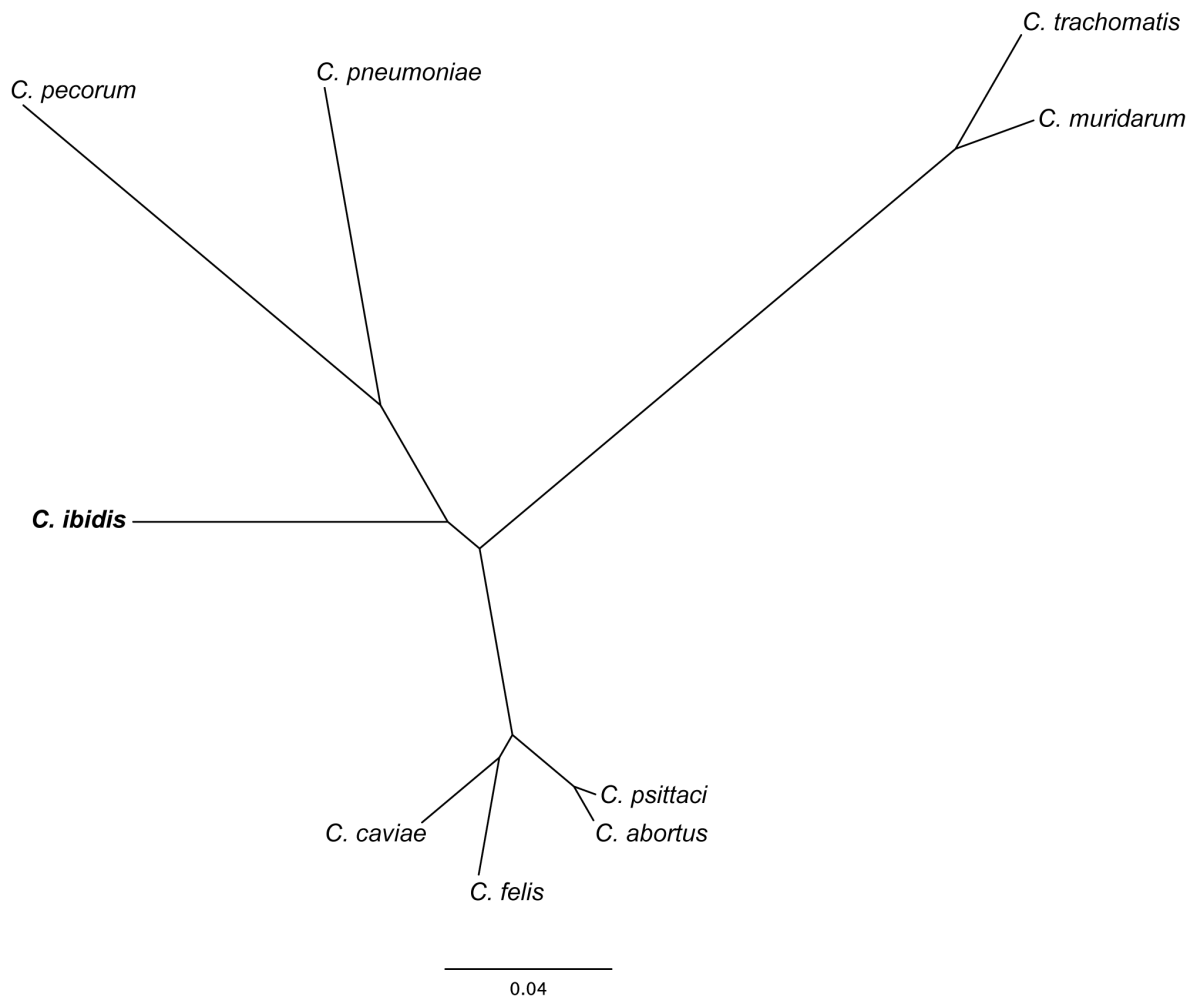
Phylogenetic tree of 31 conserved genes including corresponding sequences of *C* *. caviae* *, C. felis*, *C. psittaci,* *C* *. abortus* *,* *C* *. pecorum* *, C. pneumoniae, C. muridarum, C. trachomatis* and 10-1398/6 *C*. *ibidis* isolate. The 31 conserved housekeeping genes were concatenated, and Amphora alignments used to generate a maximum likelihood phylogeny using the PhyML implementation of the WAG model of amino acid substitution. 100 bootstrap replicates were generated.

**Figure 4:**
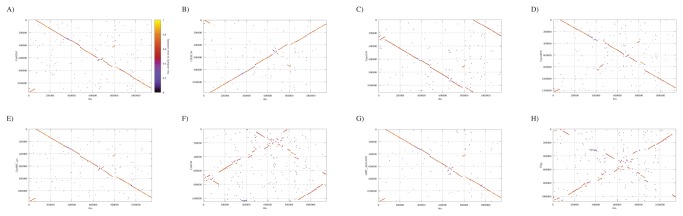
Comparison of genome structure of *C* *. ibidis* 10-1398/6 and all other members of the family *Chlamydiaceae*, specifically: A) *C* *. abortus* S263, B) *C. felis* FEC56, C) *C* *. pecorum* E58, D) *C. pneumoniae* AR39, E) *C. psittaci* 6BC, F) *C. trachomatis* D/UW-3/CX, G) *C* *. caviae* GPIC and H) *C. muridarum* Nigg. Each dot represents a peptide sequence, with color coding corresponding to the degree of similarity based on BSR.

**Figure 5:**
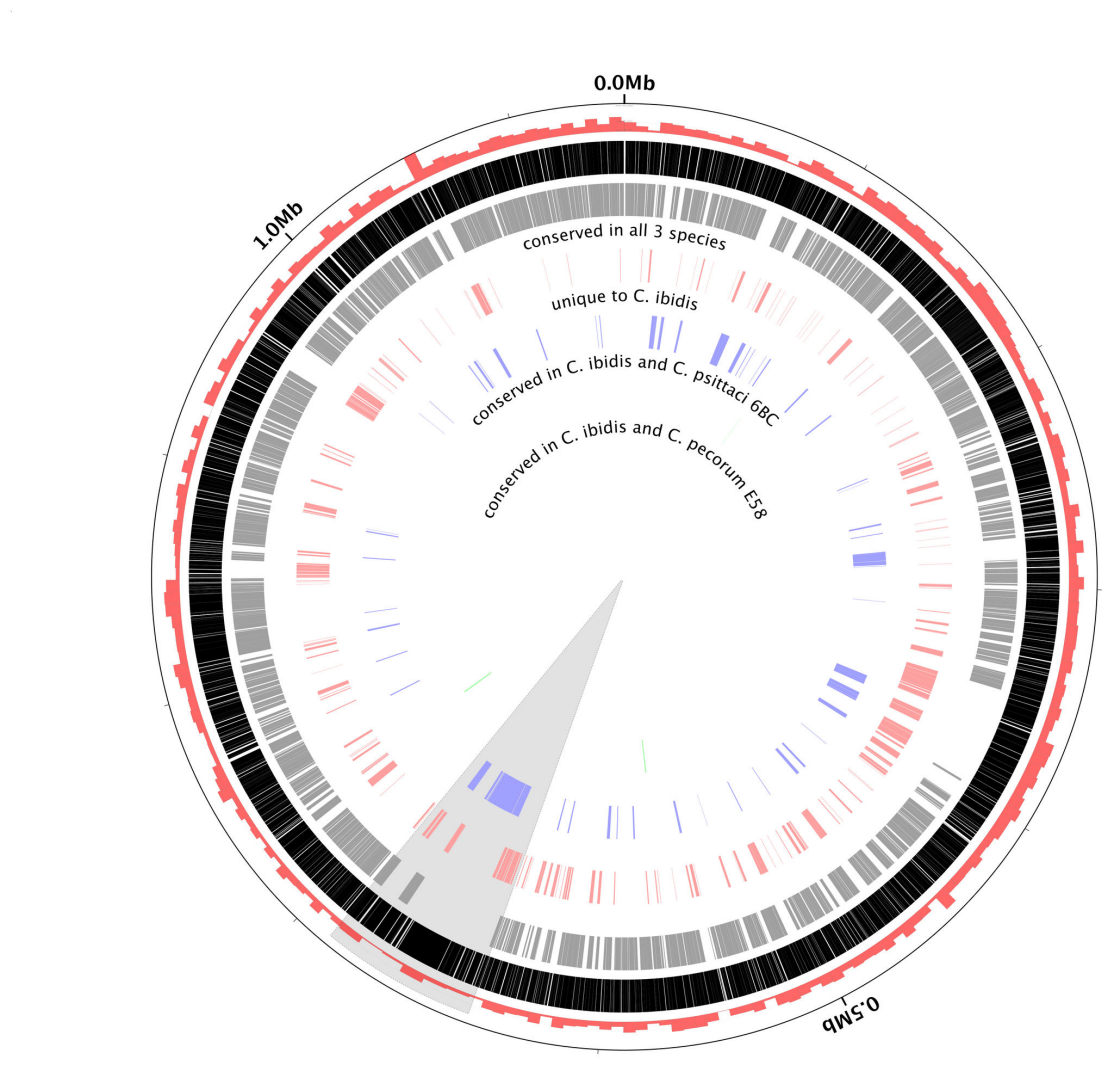
Circular representation of the 10-1398/6 genome compared to *C. psittaci* 6BC and *C* *. pecorum* E58 genomes. Outermost to innermost tracks represent: (i) % GC; (ii) all genes in 10-1398/6 (black); (iii) core genes shared by all three genomes, based on a BSR similarity >0.5 (grey); (iv) unique genes to 10-1398/6 based on a BSR similarity of <0.4 (red); (v) variable genes shared by 10-1398/6 and 6BC but absent from E58 (blue); (vi) variable genes shared by 10-1398/6 and E58 but absent from 6BC (green). Track iv (red) illustrates the presence of clusters of unique genes spanning the entire 10-1398/6 genome. The chlamydial plasticity zone is highlighted in grey.

**Figure 6:**
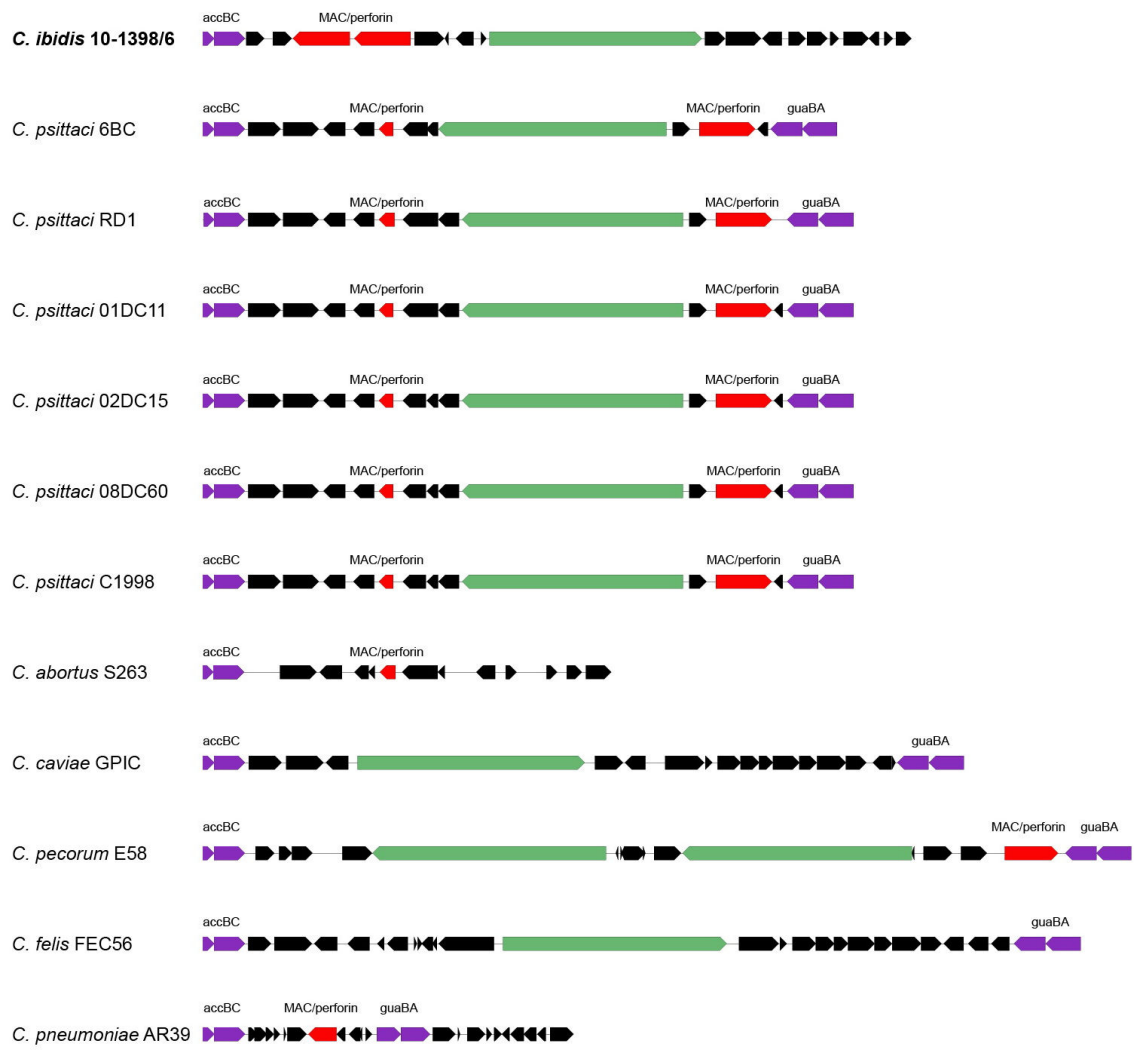
Comparison of the plasticity zones of *C. psittaci* and 10-1398/6 Ibis isolate, along with other representatives of the former genus *Chlamydophila* .

To further confirm the unique taxonomic position of isolates 10-1398/6 and 10-1398/11, genomic DNA from isolate 10-1398/6 was subjected to whole genome sequencing, assembly and annotation. The draft genome scaffold of the 10-1398/6 isolate consists of a single circular chromosome of 1,146,174 bp, encoding 1,057 predicted coding sequences (%GC content= 38.5%; average gene length= 970 bp; percent coding= 88.5%). 31 highly conserved genes were selected from the draft annotated assembly and aligned against the matching genes from all other members of the *Chlamydiaceae* family using Amphora, as described elsewhere [20]. Figure **3** displays a maximum-likelihood dendrogram based on these alignments that supports the distinctness of 10-1398/6 from these 9 established chlamydial species. Genome synteny analysis shows that the 10-1398/6 genome is similar to *C. psittaci* but with several regions of rearrangement (Figure **4**). BSR analysis of the 10-1398/6 genome compared to six *C. psittaci* genomes (including 3 avian and 3 mammalian isolates) shows that 231/1057 of the 10-1398/6 genes are unique to that genome and not shared with the other *C. psittaci* isolates (Table **2**). Many of these unique genes are found in clusters around the chromosome (Figure **5**). Comparison of the 10-1398/6 plasticity zone relative to several *C. psittaci* genomes further highlights the divergence of the 10-1398/6 isolate from *C. psittaci*, as well as the relationship to the plasticity zone of other members of the old genus *Chlamydophila* (Figure **6**).

**Table 2:** Breakdown of predicted protein orthologs between *Chlamydia psittaci* isolates and 10-1398/6 based on BSR analysis.

	# of orthologs				
**Species**	**Host**	**Core (>0.5**)	**Variable (<0.5 and >0.4**)	**Unique (<0.4**)	**Total**
*Chlamydia psittaci* 6BC	Bird	736	257	13	1006
*Chlamydia psittaci* RD1	Bird	735	215	3	953
*Chlamydia psittaci* 01DC11	Pig	738	240	0	978
*Chlamydia psittaci* 02DC15	Cattle	740	238	1	979
*Chlamydia psittaci* 08DC60	Human	738	240	0	978
*Chlamydia psittaci* C19/98	Sheep	739	239	0	978
**10-1398/6**	**Bird**	**802**	**23**	**231**	**1057**

## Discussion

Although the importance of *C. psittaci* as the causative agent of avian chlamydiosis in psittacine birds and domestic fowl has been known for decades [25], several studies provide recent evidence of the occurrence of other chlamydial species in birds, such as *C* *. abortus* [26,27], *C* *. suis* and *C. muridarum* [28] as well as *C* *. pecorum* and *C. trachomatis* [12]. Additionally, new and hitherto non-classified chlamydial agents were recently described in chickens [6,16,29], pigeons [11,12], and sea gulls [10].

Avian chlamydiosis due to *C. psittaci* is a major concern in the French duck industry where human cases of psittacosis are regularly reported. The means of contamination and dissemination of the infection in duck flocks are poorly understood. As Ibises were repeatedly observed to feed on open-air duck breeding premises [13], investigations were conducted in order to assess the occurrence of chlamydiae in these birds. Examination of cloacal swabs collected from wild Ibises showed that 11% had excreted bacteria belonging to the family *Chlamydiaceae* (Table **1**). When re-tested with a *C. psittaci*-specific real-time PCR, only one of the 8 positive Ibis specimens was positive: specimen 10-1398/28. Successful cultivation of 3 isolates (10-1398/6, 10-1398/11 and 10-1398/28) in chicken eggs allowed for a more complete examination.

Isolate 10-1398/28 was genotyped as *C. psittaci* by real-time PCR and by 16S rRNA gene sequencing. BLAST analysis of the *omp*A sequence revealed high homology with *omp*A of genotype C of *C. psittaci*. This genotype is commonly associated with waterfowl [30]. Although the dataset presented in this study is small, our results suggest a low prevalence of *C. psittaci* in the Ibis population, such that it probably does not represent a significant reservoir of the pathogen, nor a high risk for the contact duck flocks. Moreover, the detection of *C. psittaci* genotype C in one of the specimens suggests that the pathogen may have been transmitted to the Ibis from ducks, where it is relatively abundant.

Interestingly, the analysis of the 16S rRNA gene sequence of the two other Ibis isolates (10-1398/6 and 10-1398/11) suggested that they are members of the family *Chlamydiaceae*, as the 16S rRNA gene sequence homology family-level cut-off values stand at 90% [31], but suggested also that they correspond to a new and hitherto non-classified chlamydial species. Indeed, the 16S rRNA gene sequence homology between the two atypical isolates and *C. psittaci* or *C* *. abortus* was lower than that between *C* *. abortus* and *C. psittaci* (respectively 97.4% and 97.2% versus 99.6%) (Table **S2**). Phylogenetic analysis of 31 concatenated housekeeping genes from the annotated draft genome of the 10-1398/6 isolate further supports this distinct position (Figure **3**). Genome synteny analysis shows that the 10-1398/6 genome is very similar to that of *C. psittaci* but with several regions of rearrangement, and BSR analysis of the 10-1398/6 genome compared with other representative *C. psittaci* genomes revealed that 20% of the 10-1398/6 genes are unique to that genome and not shared with other *C. psittaci* isolates. This is unusual in the otherwise highly conserved *Chlamydiaceae* [32]. Analysis of the plasticity zone, a region of genetic divergence amongst the *Chlamydiaceae*, revealed a unique organization for 10-1398/6. Thus, we propose that 10-1398/6 and the highly similar 10-1398/11 correspond to a new member species of the *Chlamydiaceae*, with the proposed name *Chlamydia* *ibidis* . Studies of isolate 10-1398/6 by transmission electron microscopy confirmed this assessment. Typical membrane-bound inclusions were observed that contained EBs, RBs and intermediate developmental forms typical of the *Chlamydiaceae*. A unique distinguishing feature was the apparent lobar structure of the condensed nucleoid in EBs.

The question of the origin of *C* *. ibidis* in the Sacred Ibis colony of western France looms large. Two possibilities should be considered. First this new species may correspond to a strain that is endogenous to the local avian population yet has escaped detection to this day, perhaps because of not being sought after actively in the past or with the appropriate probes (most detection attempts usually target *C. psittaci*-specific genes or antigens). This may also be because this strain is relatively innocuous in avian species, including domesticated birds, or because it is not associated with symptomatic zoonotic infection as are its *C. psittaci* counterparts. Another tantalizing possibility is that the *C* *. ibidis* isolate is native – and perhaps specific - to Ibis birds and that it was brought over with these birds from African countries where native Ibises are prevalent. These various possibilities are now being explored.

Follow-up studies are needed in order to assess potential seasonal changes in the prevalence of *Chlamydia* among the Ibis population. Although a small data set, the number of infected birds seems to be higher in July 2010 than in March 2009. It is unclear whether this reflects accidental or seasonal changes. Seasonal fluctuation has been reported for pigeons [33,34,12]. Further studies are also needed to fully characterise the virulence properties of *C* *. ibidis* , to identify the geographical origin of the infection and its range, and to address the critical question of the health risk to humans or other animals, including poultry and avian wildlife. In the absence of this important information, individuals handling Sacred Ibises should assume that the zoonotic risk is similar to that of *C. psittaci*, and take appropriate precautionary measures.

### Description of *Chlamydia* *ibidis* sp. nov.

*Chlamydia* *ibidis* (ibidis. L. fem. gen. pl. *ibidis*, of the Ibis, because this bird is the only host currently known). The strains of this species occur in the Ibis and possibly also in other birds. The agent can be recovered from cloacal swabs and faeces. So far, no evidence of a pathogenic potential has emerged. Like all other species of the *Chlamydiaceae*, *C* *. ibidis* strains can be grown in cell culture and in embryonated chicken eggs. The morphology as revealed by electron microscopy harbors the typical features of *Chlamydia* spp., i.e. intracellular vacuolar inclusions contain electron-dense elementary bodies of less than 1 µm diameter and the slightly larger and more electron-lucent reticulate bodies. Identification of bacteria of this new species can be obtained based on its 16S rDNA and *omp*A gene sequences. The type strain is 10-1398/6^T^.

## Supporting Information

Figure S1 **Dendrogram based on the analysis of the nearly complete *omp*A gene sequences (about 970 nt) from the ibis isolates (10-1398/6 and 10-1398/11) and from the type strains of nine members of the *Chlamydiaceae*.** The dendrogram was constructed by the neighbour-joining method from phylogenetic distances calculated by UPGMA method. Bootstrap test was for 1000 repetitions. Horizontal distances correspond to genetic distances expressed in percentage of sequence similarity, vertical distances are arbitrary.
(TIF)

Table S1 **Characteristics of sequencing primers used in this study.**
(DOC)

Table S2 **Sequence similarities (%) for 16S rRNA genes of the new chlamydial isolates described in this study in comparison to the currently defined species of *Chlamydiaceae*.** Similarity values were calculated from distance matrices of nearly full-length 16S rRNA genes (about 1350 nt).
(DOC)
