# Supplementary figures and images for: Isolation of a New Chlamydia species from the Feral Sacred Ibis (Threskiornis aethiopicus): Chlamydia ibidis
Source: PLoS One. 2013 Sep 20;8(9):e74823. doi: 10.1371/journal.pone.0074823 (PMC3779242; doi:10.1371/journal.pone.0074823)

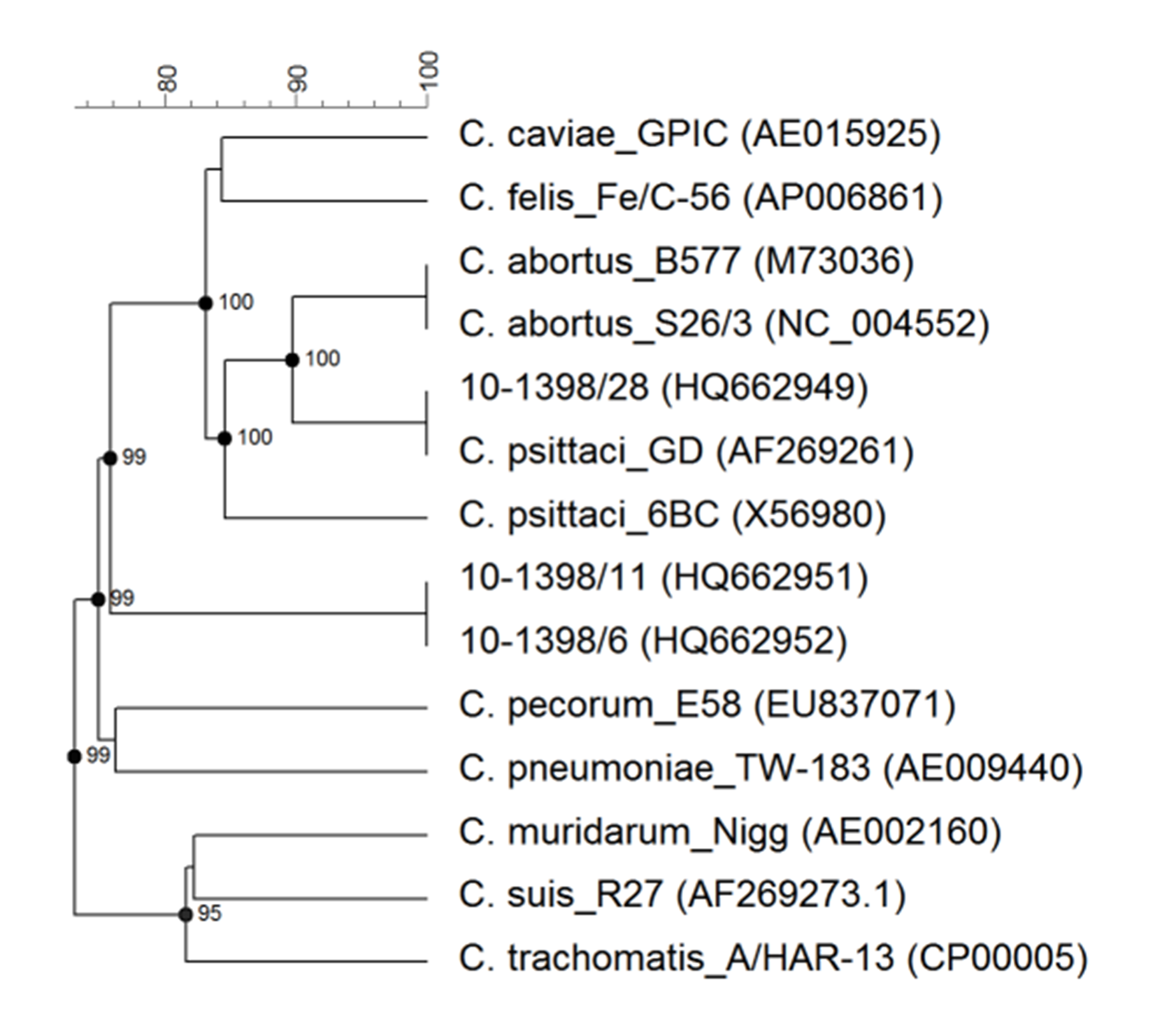

Supplement: Figure S1 — Dendrogram based on the analysis of the nearly complete ompA gene sequences (about 970 nt) from the ibis isolates (10-1398/6 and 10-1398/11) and from the type strains of nine members of the Chlamydiaceae. The dendrogram was constructed by the neighbour-joining method from phylogenetic distances calculated by UPGMA method. Bootstrap test was for 1000 repetitions. Horizontal distances correspond to genetic distances expressed in percentage of sequence similarity, vertical distances are arbitrary. (TIF) [file pone.0074823.s001.tif]
