# Supplementary material for: Isolation of a New Chlamydia species from the Feral Sacred Ibis (Threskiornis aethiopicus): Chlamydia ibidis
Source: PLoS One. 2013 Sep 20;8(9):e74823. doi: 10.1371/journal.pone.0074823 (PMC3779242; doi:10.1371/journal.pone.0074823)
Supplement: Table S1 — Characteristics of sequencing primers used in this study. (DOC) [file pone.0074823.s002.doc]

**Supplement 1**. Characteristics of sequencing primers used in this study

| **Targeted sequence** | **Primer name** | **Sequencea (5’-3’)** | **PCR product size** | **Reference** |
| --- | --- | --- | --- | --- |
| 16S rRNA | 16S1 | CGGATCCTGAGAATTTGATC | ~ 1400 bp | Pudjatmoko et al., 1997 |
|  | rp2 | CTACCTTGTTACGACTTCAT |  | Thomas et al., 2006 |
| *omp*A | ompA-forw | ACTCTTCTATGAGGGTAATTCCAACTTATT | ~ 2000 bp | This study |
|  | ompA-rev | TTTGGATTCCATCTTCGGGTT |  |  |
|  | 5GPF | acgcatgcaagacactcctcaaagcc | ~ 1200 bp | Kaltenboeck et al., 1993 |
|  | CTL | CAAGATTTTCTAGATTTCATCTTGTT |  | Denamur et al., 1991 |
