# Supplementary material for: Isolation of a New Chlamydia species from the Feral Sacred Ibis (Threskiornis aethiopicus): Chlamydia ibidis
Source: PLoS One. 2013 Sep 20;8(9):e74823. doi: 10.1371/journal.pone.0074823 (PMC3779242; doi:10.1371/journal.pone.0074823)
Supplement: Table S2 — Sequence similarities (%) for 16S rRNA genes of the new chlamydial isolates described in this study in comparison to the currently defined species of Chlamydiaceae. Similarity values were calculated from distance matrices of nearly full-length 16S rRNA genes (about 1350 nt). (DOC) [file pone.0074823.s003.doc]

**Supplement 2.** Sequence similarities (%) of 16S rRNA gene (nearly full-length, about 1350 nt) from pairwise comparison of the new chlamydial isolates described in this study and the currently defined species of *Chlamydiaceae*. *Waddlia chongrophila* was included as outgroup.
